# Risk prediction of PLA2R-Ab-negative membranous nephropathy: an interpretable multicenter machine learning model

**DOI:** 10.3389/fimmu.2026.1906174

**Published:** 2026-09-01

**Authors:** Keyan Qian, Hongfeng Niu, Yingzi Li, Fuhan Yang, Zhuolin Shi, Yongping Dang, Yuanhao Li, Jiahong Guo, Xinfang Li, Jin Li

**Affiliations:** 1 Xinxiang Key Laboratory of Microfluidic Immunodiagnosis for Kidney Diseases, Kidney Disease Hospital, The First Affiliated Hospital of Henan Medical University, Henan Province, Xinxiang, China; 2 Network and Information Center, The First Affiliated Hospital of Henan Medical University, Henan Province, Xinxiang, China; 3 Department of General Practice, The First Affiliated Hospital of Henan Medical University, Henan Province, Xinxiang, China; 4 Department of Nephrology, Xinxiang Central Hospital, Henan Province, Xinxiang, China; 5 Department of Nephrology, Xinhua Hospital, Henan Province, Anyang, China

**Keywords:** anti-phospholipase A2 receptor antibody, machine learning, membranous nephropathy, noninvasive testing, risk stratification performance

## Abstract

**Background:**

Approximately 10%–30% patients with biopsy-proven membranous nephropathy (MN) are seronegative for anti-phospholipase A2 receptor antibody (PLA2R-Ab). Only ~5% are truly non-PLA2R MN and are associated with alternative antigens (e.g., THSD7A), while the majority represent false-negative PLA2R-associated MN due to insufficient assay sensitivity. Accurate noninvasive diagnostic tools for this population are lacking.

**Methods:**

This multicenter retrospective risk stratification study enrolled 692 PLA2R Ab negative patients in the derivation cohort and 333 patients in an independent external validation cohort. We developed and validated an interpretable VotingSoft machine learning model based on 13 key clinical variables.

**Results:**

The model achieved excellent risk stratification performance: AUC 0.878 (95% confidence interval (CI): 0.850–0.903) in five-fold cross-validation, 0.894 (95% CI: 0.854–0.930) in the internal test set, and 0.905 (95% CI: 0.867–0.935) in the external validation cohort. The model showed a specificity of 0.819, sensitivity of 0.764, and negative predictive value of 0.876. Key predictors were PLA2R Ab level, eGFR, age, and serum albumin.

**Conclusion:**

This multicenter interpretable machine learning model provides an auxiliary tool for risk stratification of PLA2R-Ab-negative membranous nephropathy (MN) using routine clinical indicators. Its high specificity may facilitate early risk stratification; however, renal biopsy remains the gold standard for definitive pathological diagnosis.

## 1 Introduction

MN is a leading cause of adult-onset nephrotic syndrome (1) and accounts for 20%–30% of primary glomerular diseases worldwide (2, 3). Its pathological hallmarks include diffuse glomerular basement membrane thickening (4) and subepithelial immune complex deposition (5), which commonly lead to massive proteinuria, hypoalbuminemia, progressive renal function decline, and even end-stage renal disease (6, 7). Thus MN imposes a heavy clinical and socioeconomic burden.

The discovery of anti-M-type phospholipase A2 (8) receptor (9, 10) autoantibodies represents a landmark advancement in MN risk evaluation. With high sensitivity (70%–80%) (11–13) and specificity (>95%) (14, 15), PLA2R-Ab testing enables noninvasive identification of seropositive patients (16) and reduces unnecessary kidney biopsies and related procedural risks (17). However, 10% to 30% of patients with biopsy-confirmed MN are seronegative for PLA2R-Ab (serum titer ≤14 RU/mL) (15, 18–20). This subgroup comprises two distinct entities: only approximately 5% represent true non-PLA2R MN associated with alternative antigens (21) such as THSD7A (22, 23), whereas the majority represent false-negative PLA2R-associated MN. This difference is largely attributable to the limited sensitivity of current serological assays, which have a high diagnostic threshold and are unable to detect antibodies against linear epitopes (24, 25). As a result, these patients rely on invasive renal biopsy for confirmation, leading to avoidable risks and additional medical costs (26). Clinical guidelines highlight an urgent need for noninvasive tools for this underserved population.

In recent years, machine learning (ML) has been increasingly used to develop predictive models for glomerular diseases, owing to its strong ability to capture complex nonlinear relationships in high-dimensional clinical data (27–29). Most ML diagnostic models for MN have focused on PLA2R-Ab-positive cohorts or mixed populations, whereas few studies have specifically targeted the PLA2R-Ab-negative subgroup. Conventional statistical models and single biomarkers offer limited accuracy and generalizability in this clinically challenging population, leaving a critical risk prediction gap. Meanwhile, many ML models are limited by poor interpretability—the so-called “black box” problem—which hinders clinical acceptance and translation (30). To address this, SHapley Additive exPlanations (SHAP) has been used to open the black box of ML models in nephrology, providing transparent and intuitive explanations of predictive logic and enhancing clinician trust. However, few studies have combined high original diagnostic performance with rigorous interpretability and external validation for PLA2R-Ab-negative MN. Furthermore, there is a lack of freely accessible, easy-to-use, noninvasive auxiliary tools for rapid risk stratification in routine nephrology practice, making clinical translation of predictive models unsatisfactory.

Against this background, we conducted this multicenter retrospective risk stratification study with the following objectives: (1) the primary objective was to develop and externally validate an interpretable ML model using routine clinical and laboratory data to improve identification of PLA2R-associated MN among PLA2R-Ab-negative proteinuric patients by distinguishing them from other common proteinuric kidney diseases; (2) the secondary objectives were to identify the optimal algorithm–feature selection combination with the best discrimination, specificity, and stability, to interpret the model’s predictive mechanism using SHAP to enhance clinical transparency and adoption, and to deploy the model as a free, user-friendly web application to provide auxiliary risk stratification reference (31).

## 2 Methods

### 2.1 Research design and data sources

This multicenter retrospective risk stratification study was conducted in three hospitals in Henan Province, Central China: The First Affiliated Hospital of Henan Medical University (tertiary, derivation cohort), Xinxiang Central Hospital (tertiary, external validation), and Xinhua Hospital (secondary, external validation). De-identified electronic medical records were collected from the three centers. Ethical approval was obtained from the Institutional Review Board of The First Affiliated Hospital of Henan Medical University (Approval No. EC-025-92). The study was performed in accordance with the Declaration of Helsinki (2013 revision). Written informed consent was waived due to the anonymized retrospective nature of the analysis.

### 2.2 Sample size calculation

**Figure 1:**
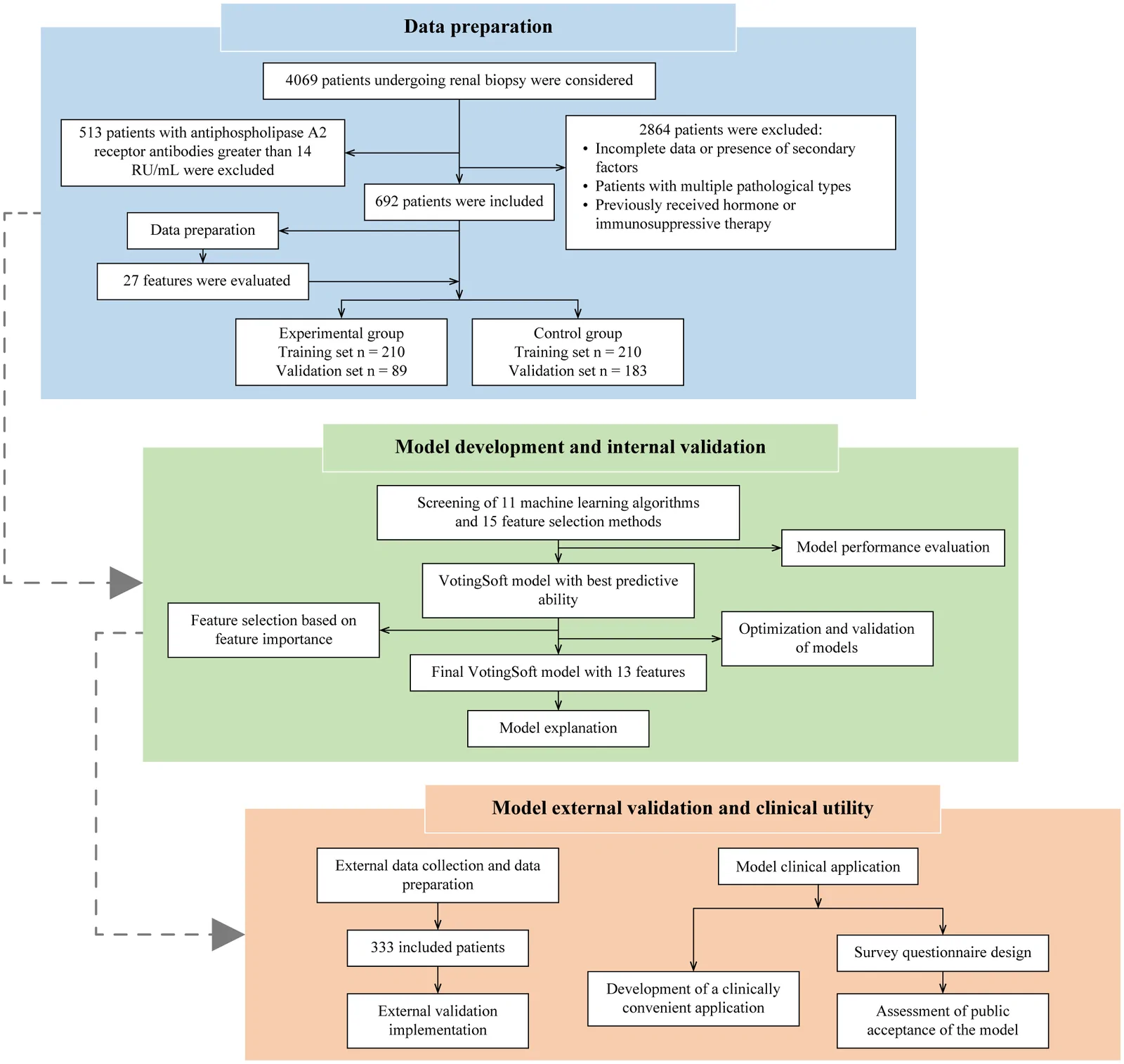
Research flowchart. The flowchart comprises three components: data preparation; model development and internal validation; and model external validation and clinical utility. A total of 4069 renal biopsy patients were screened, of whom 692 eligible PLA2R-Ab-negative patients were included in the derivation cohort after exclusions (PLA2R-Ab >14 RU/mL, secondary MN, incomplete data, or prior hormone/immunosuppressive therapy); eleven ML algorithms, combined with 15 feature selection methods, were screened, and the optimal VotingSoft model (13 key features), was selected via 10-repeat five-fold cross-validation, with external validation conducted in an independent cohort (n = 333).

Sample size was calculated based on the 10 events per predictor (EPP) rule for predictive modeling. The final model included 13 candidate predictors, requiring at least 130 events (MN cases) to ensure statistical stability. The present study included 210 MN patients and 210 matched controls in the training set, which exceeded the minimum requirement. *Post hoc* power analysis was performed based on an AUC of 0.894 (type I error α=0.05, test power=0.80), confirming that the final statistical power was greater than 90%, which was sufficient for discriminative model evaluation. The study flowchart is presented in **Figure 1**.

### 2.3 Study cohort and inclusion criteria

Eligible participants were adults aged ≥18 years who underwent kidney biopsy between January 2018 and September 2025. The case group consisted of patients with biopsy-proven primary MN and a serum PLA2R-Ab titer ≤14 RU/mL (manufacturer-defined negative threshold). The control group included patients with biopsy-proven diabetic nephropathy (DN), minimal change disease (MCD), or IgA nephropathy (IgAN), also with PLA2R-Ab ≤14 RU/mL. Exclusion criteria were: (1) secondary MN; (2) concurrent other primary glomerular diseases; (3) incomplete key data within 48 hours of admission; (4) prior kidney transplantation.

### 2.4 Data collection, preprocessing, and feature engineering

A standardized case report form was used to extract 32 candidate predictor variables, including demographics, vital signs, biochemistry, coagulation, and urinalysis parameters. All variables were obtained within the first 48 hours of admission.

Data preprocessing used the following protocol:

- Missing value handling: Variables with missing rates >30% were excluded. Variables with missing rates between 10% and 30% were imputed by multiple imputation by chained equations (MICE) with 10 iterations and predictive mean matching (32). Variables with missing rates <10% were imputed using median imputation for skewed data and mean imputation for normally distributed data (33). The missing rate of all included variables was <30%, and no key variable was excluded due to excessive missingness.
- Correlation and redundancy reduction: Spearman correlation coefficients were calculated between continuous variables. If the correlation coefficient exceeded 0.7, the variable with weaker univariate association with the outcome (MN vs non-MN) was removed (**Supplementary Figure 1**).
- Feature scaling: All continuous features were standardized using z-score normalization (StandardScaler) to achieve a mean of 0 and a standard deviation of 1.

After preprocessing, 27 core features, including age, weight (Weight), PLA2R-Ab, 24-hour urinary total protein (U-TP), albumin (ALB), triglyceride (TG), cholesterol (CHOL), high-density lipoprotein (HDL), creatinine (CREA), sodium (Na), potassium (K), chloride (Cl), bicarbonate (HCO_3_^−^), alanine aminotransferase (ALT), aspartate aminotransferase (AST), alkaline phosphatase (ALP), eGFR, high-sensitivity C-reactive protein (HsCRP), white blood cell (WBC), red blood cell (RBC), platelet (PLT), prothrombin time (PT), activated partial thromboplastin time (APTT), D-dimer (D-D), fibrinogen (FIB), urinary red blood cell count (U-RBC), and urinary white blood cell count (U-WBC), were retained for model development.

### 2.5 Model development, optimization, and selection

The derivation cohort (n=692; 299 MN, 393 non-MN) was randomly split into training and internal test sets in a 7:3 ratio using stratified sampling to preserve class balance. MN patients were assigned to training (n=210, 70%) and internal test sets (n=89, 30%). Non-MN patients were randomly sampled to match the MN training set size (n=210) using a fixed random seed (random_state=42) to ensure reproducibility, with the remainder allocated to the internal test set (n=183). We evaluated 11 machine learning algorithms, encompassing logistic regression, ridge regression, support vector machine (SVM), Naive Bayes, random forest, extreme gradient boosting (XGBoost), LightGBM, CatBoost, gradient boosting, neural network (multi-layer perceptron), as well as a soft voting ensemble strategy (VotingSoft). Each predictive model was integrated with 15 alternative feature selection techniques, including LASSO, recursive feature elimination (RFE), mutual information, chi-square test, Boruta, and no selection, yielding 165 candidate models. Model performance was assessed using the area under the receiver operating characteristic curve (AUC), accuracy, sensitivity, specificity, positive predictive value (PPV), negative predictive value (NPV), and F1-score. Stability was evaluated using 10-repeated 5-fold cross-validation. The optimal model was defined as the combination with the highest mean AUC and lowest standard deviation across folds.

### 2.6 Model interpretation and feature importance

Model interpretability was realized using the SHapley Additive exPlanations (SHAP) framework (SHAP v0.49.1, Python 3.12). For each prediction in the internal test set, SHAP values were computed to derive:

- Global interpretability: The mean absolute SHAP value per feature was used to rank overall feature importance. SHAP summary and dependence plots were produced to illustrate the direction and magnitude of each feature’s influence on model outputs.
- Local interpretability: For individual patient predictions, waterfall plots were generated to delineate how each feature contributed to shifting the baseline prediction probability toward the final outcome, thereby providing a case-specific “reasoning trail.”

### 2.7 Validation strategy

- Internal validation: The performance of the final model was assessed on the held-out internal test set from the derivation cohort; all data were processed via the identical preprocessing pipeline to evaluate performance within the same patient population.
- External validation: The final model, fixed without any retraining, was used to test the independent external cohort from two different hospitals (XCH and XHH). This was processed using the identical preprocessing pipeline derived from the training set, to evaluate generalizability across different clinical centers and patient populations.
- Clinical utility assessment: Decision curve analysis (DCA) was performed across a spectrum of threshold probabilities (0.1 to 0.9) to quantify the net clinical benefit of applying the model to guide biopsy decisions, in comparison to “biopsy-all” or “biopsy-none” approaches.

### 2.8 Statistical analysis

Continuous variables were expressed as median (interquartile range) and compared using the Mann–Whitney U test. Categorical variables were presented as frequencies (%) and compared using the chi-square test. All statistical comparisons were two-sided, and P<0.05 was considered statistically significant. Restricted cubic spline (RCS) analysis with 3 knots (default clinical practice setting) was used to explore nonlinear relationships between PLA2R-Ab levels and MN risk. Knots were placed at the 10th, 50th, and 90th percentiles using the R software (version 4.5.0) with the ‘rms’ package. All statistical modeling was performed in Python 3.12 using scikit-learn, XGBoost, LightGBM, and SHAP libraries.

## 3 Results

### 3.1 Study population and baseline characteristics

The derivation cohort comprised 692 patients from the First Affiliated Hospital of Henan Medical University, including 299 patients with membranous nephropathy (MN) and 393 patients with other proteinuric kidney diseases (non-MN: 221 diabetic nephropathy, 98 minimal change disease, 74 IgA nephropathy). An independent external validation cohort of 333 patients was recruited from Xinxiang Central Hospital and Xinhua Hospital (MN: n=126; non-MN: n=207). In the derivation cohort, compared with non-MN patients, MN patients were older (median age 50.0 vs. 43.0 years, P<0.001), had higher serum PLA2R-Ab (3.1 vs. 1.8 RU/mL, P<0.001), total cholesterol (6.5 vs. 5.4 mmol/L, P<0.001), and 24-hour urinary protein (2762.4 vs. 1744.5 mg/24H, P<0.001), but lower serum albumin (31.1 vs. 37.6 g/L, P<0.001) and eGFR (107.7 vs. 102.0 mL/min/1.73 m^2^, P<0.001). No significant between-group differences were observed in sex (P = 0.59), weight (P = 0.75), hemoglobin (P = 0.75), white blood cell count (P = 0.09), platelet count (P = 0.34), prothrombin time (P = 0.41), or activated partial thromboplastin time (P = 0.99) (**Table 1**).

**Table 1:** Comparison of demographic and clinical characteristics between patients with MN and non MN in the derivation cohort.

Variables	MN (n=299)	Non-MN (n=393)	*P* value
Age, years	50·0[40·0, 57·0]	43·0[31·0,55·0]	**<0·001**
Male, n (%)	156·0 (52·2)	197·0 (50·1)	0·59
Weight, kg	69·5[61·0,80·0]	70·0[60·0,80·0]	0·75
PLA2R-Ab, RU/mL	3·1[1·9,6·1]	1·8[1·2,2·6]	**<0·001**
Hb, g/L	134·0[122·0,147·0]	133·0[118·0,149·0]	0·75
WBC, 10^9^/L	6·3[5·1,7·4]	6·5[5·3,7·8]	0·09
RBC, 10^12^/L	4·5[4·1,4·9]	4·5[4·0,4·9]	0·52
PLT, 10^9^/L	253·0[213·0,306·0]	251·0[210·0,300·0]	0·34
Na, mmol/L	141·0[139·0,143·0]	140·0[138·0,142·0]	**<0·001**
K, mmol/L	4·1[3·8,4·4]	4·2[3·9,4·4]	0·06
Cl, mmol/L	107·0[105·0,108·9]	106·0[104·3,108·1]	**<0·001**
Ca, mmol/L	2·1[2·0,2·2]	2·2[2·0,2·31]	**<0·001**
HCO_3_-,mmol/L	26·0[23·8,28·1]	25·3[23·0,27·1]	0·003
ALT, U/L	19·0[14·0,25·0]	19·0[13·0,28·0]	0·76
AST, U/L	20·0[17·0,25·0]	19·0[15·0,25·5]	0·10
ALP, U/L	63·0[52·0,77·0]	67·0[56·0,83·5]	0·007
eGFR, mL/min/1·73 m^2^	107·7[100·6,117·5]	102·0[86·4,116·7]	**<0·001**
ALB, g/L	31·1[27·0,36·8]	37·6[26·9,43·3]	**<0·001**
TG, mmol/L	1·8[1·3,2·7]	1·6[1·1,2·3]	0·004
CHOL, mmol/L	6·5[5·4,7·87]	5·4[4·4,7·3]	**<0·001**
LDL, mmol/L	3·7[2·9,4·7]	3·0[2·3,4·3]	**<0·001**
HDL, mmol/L	1·5[1·2,1·8]	1·3[1·1,1·7]	**<0·001**
CREA, umol/L	56·7[46·5,68·0]	70·7[57·6,83·3]	**<0·001**
HsCRP, mg/L	0·9[0·4,1·7]	1·1[0·5,3·0]	**<0·001**
ESR, mm/h	23·0[12·0,33·0]	23·0[10·0,34·5]	0·23
PT, sec	11·1[10·2,11·7]	10·9[10·2,11·8]	0·41
APTT, sec	31·4[27·0,36·3]	30·8[27·3,36·3]	0·99
D-D, ug/ml	0·7[0·5,0·9]	0·6[0·4,0·8]	0·009
FIB, mg/dl	369·0[325·1,444·5]	352·0[289·9,465·8]	0·03
U-RBC,/ul	12·0[4·0,29·0]	15·0[4·5,56·0]	0·02
U-WBC,/ul	5·0[2·0,9·0]	7·0[3·0,11·0]	**<0·001**
U-TP, mg/24H	2762·4[1340·4,5315·8]	1744·5[654·7,4458·1]	**<0·001**

Data are presented as median (interquartile range) for non-normally distributed continuous variables and as frequency (%) for categorical variables; group comparisons were performed using the Mann-Whitney U test (continuous variables) or chi-square test (categorical variables), with a two-sided *P* < 0.05 considered statistically significant. Note: Bold values represent statistical significance at the level of P < 0.001.

### 3.2 Model development and optimal algorithm selection

**Figure 2:**
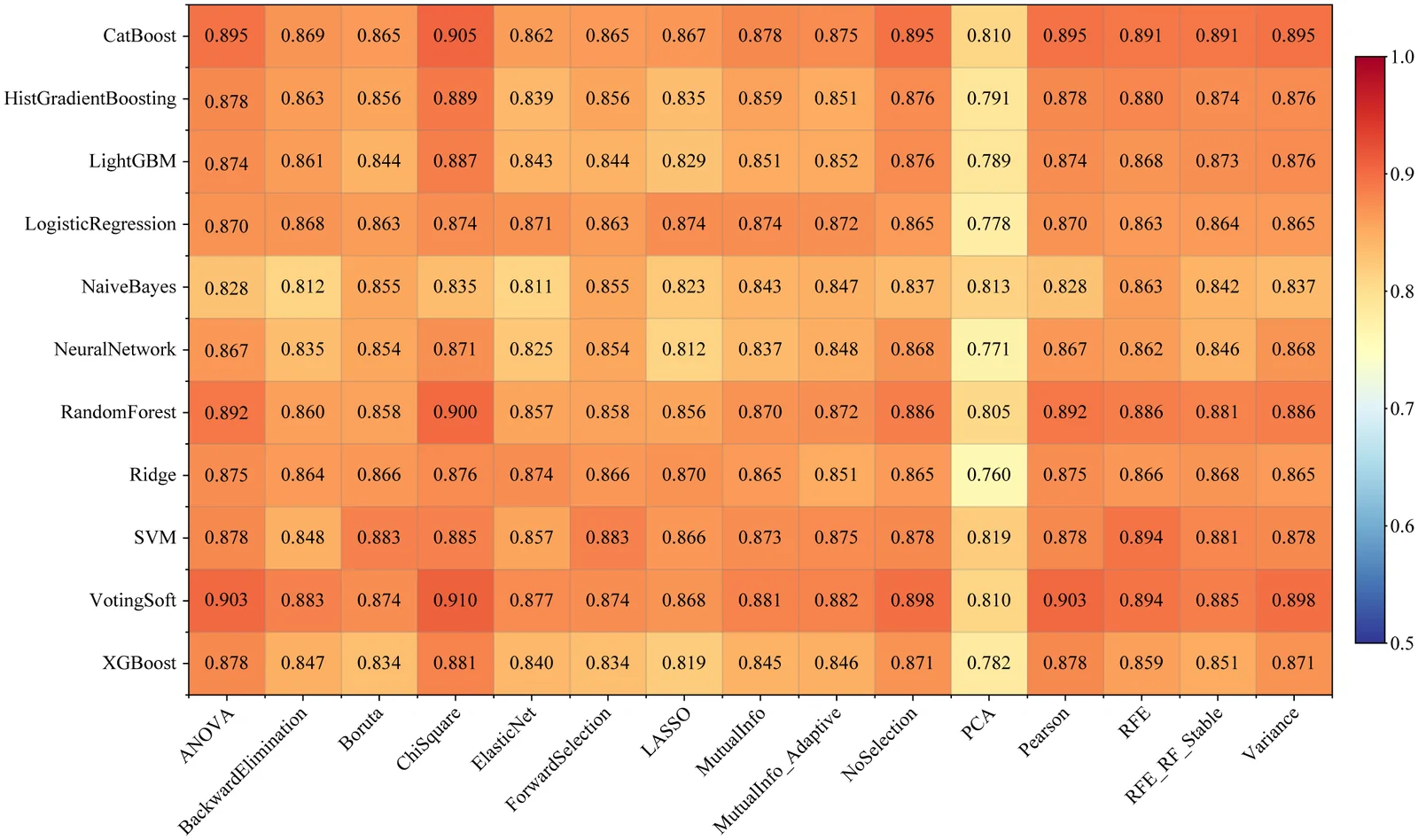
Comparison of 11 machine learning algorithms&15 feature selection methods combinations.

We used a 10-repeat design to minimize random error. The VotingSoft ensemble with chi-square feature selection achieved optimal performance (**Figure 2**): accuracy 0.836 (95% CI: 0.833–0.839), sensitivity 0.810 (95% CI: 0.802–0.818), specificity 0.848 (95% CI: 0.845–0.852), PPV 0.722 (95% CI: 0.717–0.726), NPV 0.902 (95% CI: 0.898–0.906), F1-score 0.763 (95% CI: 0.759–0.768), and AUC 0.910 (95% CI: 0.908–0.912). Narrow confidence intervals confirmed high stability. Five-fold cross-validation yielded fold-wise AUCs of 0.879, 0.868, 0.900, 0.918, and 0.825, with a mean AUC of 0.878 (95% CI: 0.850–0.903). All ROC curves performed well above random classification and clustered in the high-performance region, with minimal dispersion (SD = 0.031), indicating excellent consistency. This combination outperformed all others in both discrimination and stability.

### 3.3 Final model performance in internal and external validation

Building upon the optimal algorithm-feature selection combination, 13 pivotal features were identified via chi-square test, culminating in the development of a robust VotingSoft model for predicting PLA2R-Ab-negative MN. This model achieved an AUC of 0.894 (95% CI: 0.854–0.930), sensitivity of 0.764, specificity of 0.819, PPV of 0.673, NPV of 0.876, accuracy of 0.801, and F1-score of 0.716; the five-fold cross-validation mean AUC was 0.878 (95% CI: 0.850–0.903) (**Supplementary Data**; **Supplementary Figure 2**). Model calibration was confirmed by using a calibration intercept of −0.642 and slope of 1.175, with consistent good calibration in external validation. In comparison with the standalone PLA2R-Ab indicator (**Supplementary Data**; **Supplementary Figure 3**), at a predicted probability threshold of 0.2 (determined by the maximum Youden index for the model’s risk stratification outcome) the model yielded an NRI of −0.052 (95% CI: −0.125–0.022), with favorable reclassifications observed, demonstrating the model achieved more accurate risk reclassification for 5.2% of patients in the diagnostic gray zone. Together with the improved AUC and stable calibration, these results demonstrate the model’s improved predictive accuracy and clinical utility.

### 3.4 Subgroup analysis based on PLA2R-Ab level

**Figure 3:**
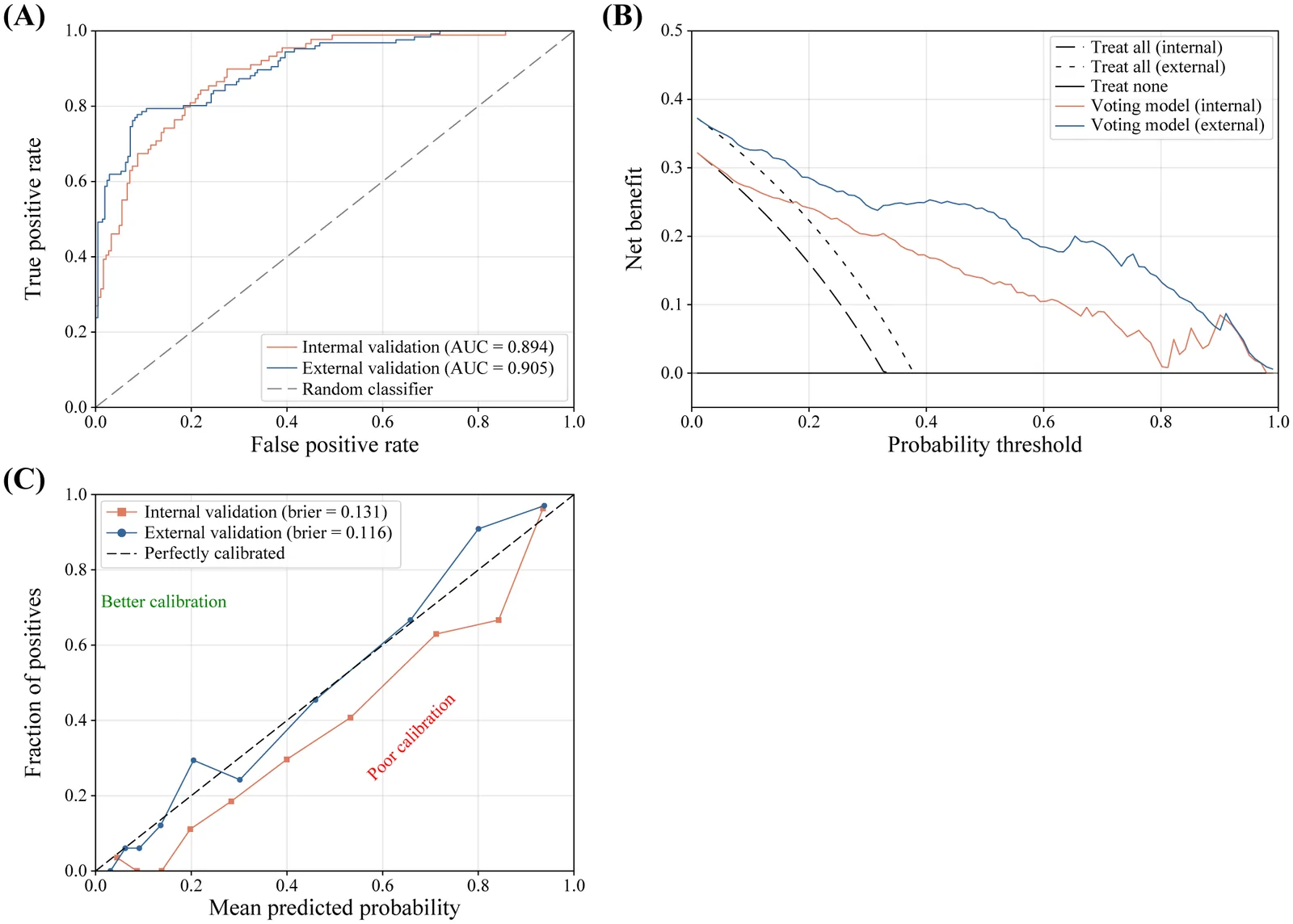
Internal and external validation of the final VotingSoft model for PLA2R-Ab-negative MN risk stratification. Panel **(A)** shows ROC curves with an AUC of 0·894 (95% CI: 0·854–0·930) for the internal test set and 0·905 (95% CI: 0·867–0·935) for the external validation cohort. Panel **(B)** presents DCA curves quantifying the net clinical benefit of the model compared with “biopsy-all” or “biopsy-none” strategies. Panel **(C)** displays the calibration curve, showing agreement between predicted and observed outcomes.

**Figure 4:**
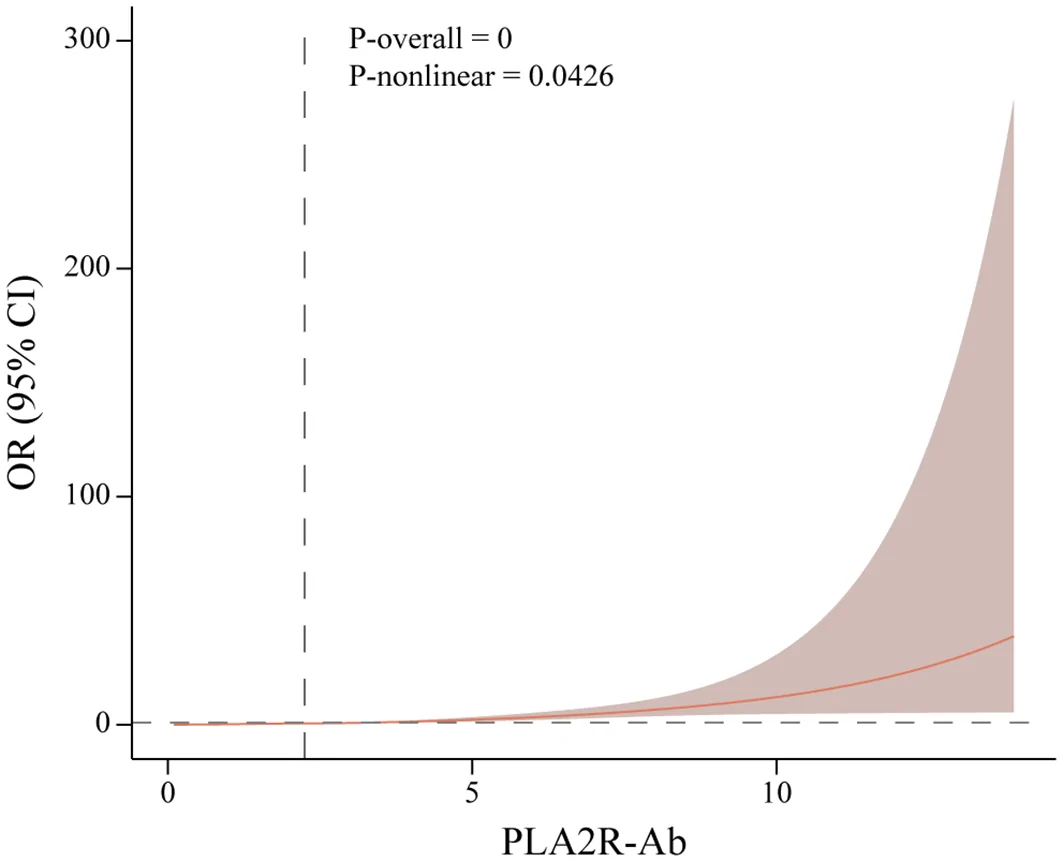
Nonlinear association between PLA2R-Ab levels and PLA2R-Ab-negative MN risk in the derivation cohort. RCS analysis with three knots revealed a nonlinear association between PLA2R-Ab levels (exposure, RU/mL) and the log-odds of MN (outcome), with an inflection point at 2·25 RU/mL; the overall association was highly significant (P-overall < 0·001) and the nonlinear component was statistically significant (P-nonlinear = 0·04), with a steeper curve above the inflection point indicating increased MN risk with rising PLA2R-Ab levels; the shaded area represents the 95% CI of the predicted log-odds.

The final model achieved an AUC of 0.905 (95% CI: 0.867–0.935) in external validation, with excellent performance in both internal and external validation (**Figure 3**). RCS analysis revealed a nonlinear association between PLA2R-Ab levels and MN risk, with an inflection point at 2.25 RU/mL (*P* for nonlinearity=0.04; **Figure 4**). To confirm the model’s discriminative ability across titer levels, the external validation cohort was stratified by this cutoff into low-titer (PLA2R-Ab ≤2.25 RU/mL) and high-titer (PLA2R-Ab >2.25 RU/mL) subgroups. In the low-titer subgroup (PLA2R-Ab ≤2.25 RU/mL, n=216), the model yielded an AUC of 0.857 (95% CI: 0.777–0.922), an accuracy of 0.884 (95% CI: 0.838–0.926), a sensitivity of 0.643 (95% CI: 0.486–0.790), and a specificity of 0.943 (95% CI: 0.903–0.973). In the high-titer subgroup (PLA2R-Ab >2.25 RU/mL, n=117), performance was notably improved, with an AUC of 0.892 (95% CI: 0.830–0.944), an accuracy of 0.812 (95% CI: 0.744–0.872), a sensitivity of 0.810 (95% CI: 0.720–0.889), and a specificity of 0.818 (95% CI: 0.683–0.936). Subgroup analysis showed improved performance in the high-titer subgroup compared with the low-titer subgroup (**Table 2**).

**Table 2:** Comparison of indications between subgroups for external validation.

Subgroup	n	AUC	Accuracy	Sensitivity	Specificity	NPV	Brier score
1	216	0·857	0·884	0·643	0·943	0·916	0·104
2	117	0·892	0·812	0·810	0·818	0·628	0·137

The external validation cohort was stratified by the PLA2R-Ab inflection point (2·25 RU/mL) identified via restricted cubic spline analysis; performance metrics include AUC, sensitivity, specificity, NPV, and Brier Score.

### 3.5 Model interpretation and feature importance

**Figure 5:**
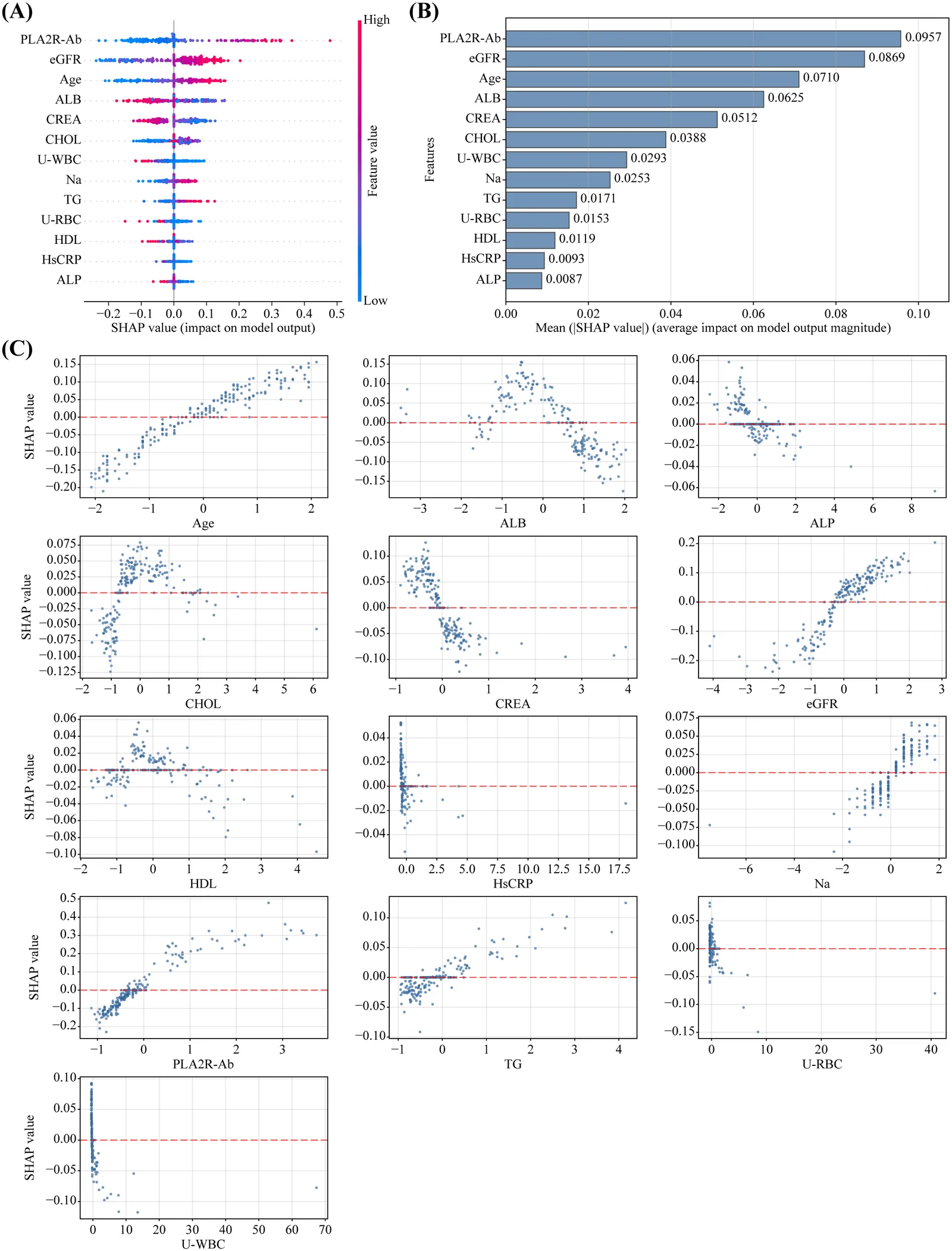
SHAP analysis for interpretation of the final VotingSoft model. Panel **(a)** displays a SHAP summary beeswarm plot, which illustrates the direction (red indicates high feature values, blue denotes low feature values) and magnitude of each feature’s influence on the model’s output; Panel **(b)** presents a SHAP summary bar chart that ranks feature importance based on the mean absolute SHAP value, identifying the PLA2R-Ab, eGFR, age, and ALB as the top four influential features; Panel **(c)** presents SHAP dependence plots illustrating nonlinear relationships between top features and predicted MN risk.

**Figure 6:**
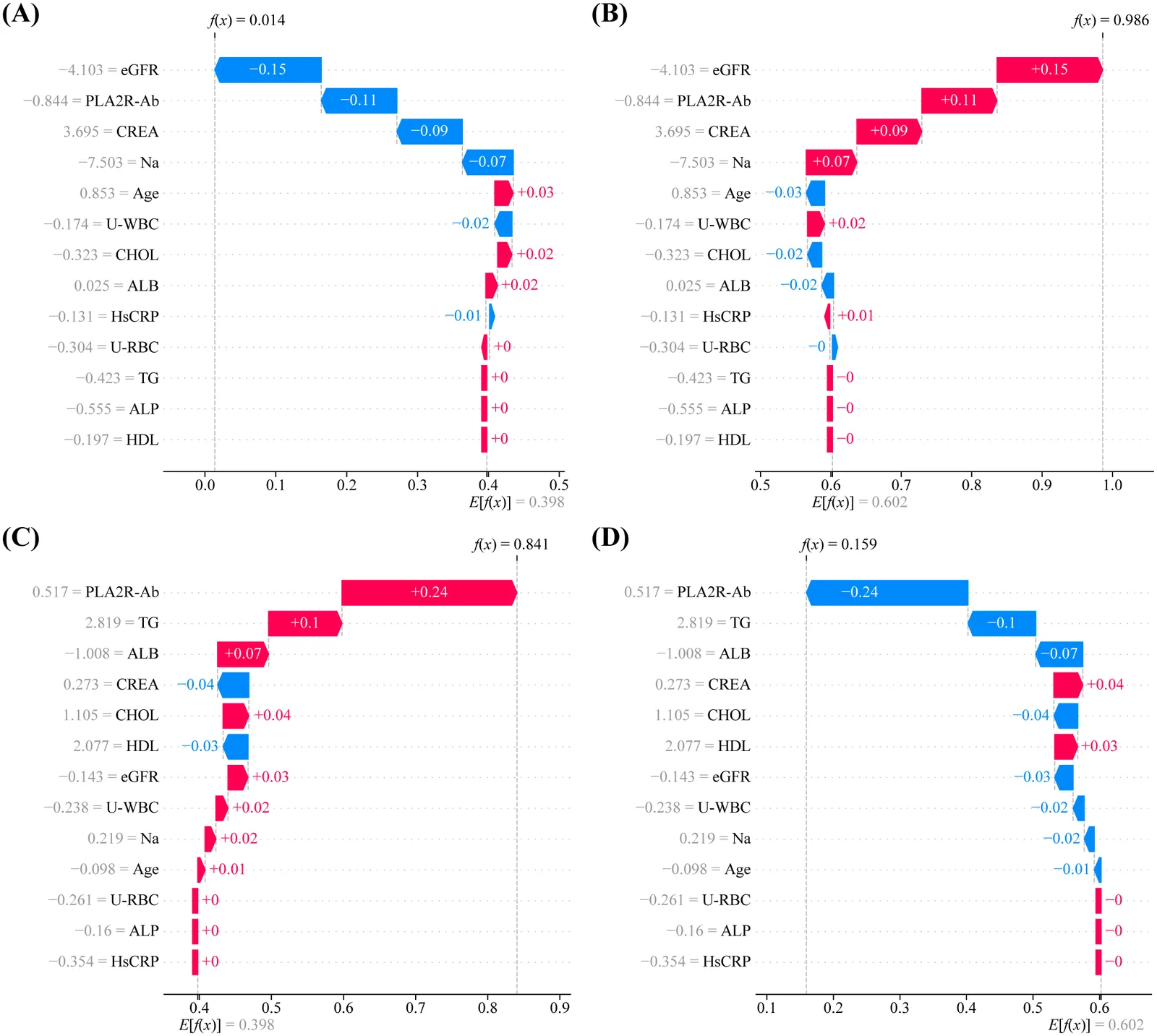
Explanation of SHAP method for local models **(a–d)**. Waterfall plot and risk evolution of each feature for low **(a, b)** or high **(c, d)** risk patients with membranous nephropathy.

SHAP analysis clarified the model’s decision logic. At a global level, the four most influential features driving MN prediction were, in descending order: PLA2R-Ab level, eGFR, patient age, and ALB (**Figures 5A, B**). SHAP dependence plots illustrated the directional impact: lower ALB, higher PLA2R-Ab (even within the “negative” range), lower eGFR, and older age were associated with increased predicted risk of MN (**Figure 5C**). At an individual level, waterfall plots provided case-specific reasoning, visually demonstrating how each feature contributed to shifting the baseline risk towards the final prediction (**Figure 6**).

### 3.6 Clinical translation: web application deployment

The final model was implemented as a freely accessible, interactive web-based tool (Supplementary data, **Supplementary Figure 4**). Clinicians can enter the 13 routinely available feature values (PLA2R-Ab, U-WBC, CREA, U-RBC, HsCRP, age, TG, eGFR, CHOL, HDL, ALB, Na, ALP) to obtain an instantaneous and interpretable prediction of the probability of PLA2R-Ab–negative MN. This output is accompanied by a visual breakdown of the contributing factors (the web application is accessible at http://11932la638ny4.vicp.fun). To assess the clinical acceptability of the model, a cross-sectional survey was conducted among 371 respondents, including nephrologists, primary care physicians, and patients with proteinuric kidney disease. Respondents were recruited from the outpatient and inpatient departments of our center using convenience sampling. After being informed of the procedural risks and limitations of renal biopsy, 77.90% (289/371) of the respondents preferred to use this noninvasive prediction tool as the initial risk stratification strategy, further supporting the model’s potential clinical utility.

## 4 Discussion

In this study, we developed and externally validated an interpretable ML model for identifying PLA2R-Ab-negative MN using 13 routine clinical variables. The VotingSoft model achieved an external validation AUC of 0.905 (95% CI: 0.867–0.935) and high specificity with favorable clinical net benefit, which delivers auxiliary risk stratification evidence for seronegative proteinuric patients, without substituting renal biopsy for final diagnosis.

Compared with existing predictive models for membranous nephropathy, our study has several key strengths. Most previous models focused on PLA2R-Ab-positive or mixed populations, whereas our model specifically targets the clinically challenging PLA2R-Ab-negative subgroup. With a large derivation cohort (n = 692) and independent external validation (n = 333), our model achieved high and stable discriminative performance. In contrast to many “black-box” machine learning approaches, our model provides full interpretability, rigorous validation, and practical clinical translation, representing a more robust and applicable tool.

The proposed model performed well in both internal and external validation, confirming that routine clinical indicators can effectively risk-stratify patients with negative PLA2R-Ab results. Importantly, we identified a nonlinear association between PLA2R-Ab levels and MN risk, with an inflection point at 2.25 RU/mL. Even within the seronegative range, values above this threshold were associated with a steeply increased risk of MN. SHAP analysis revealed that predictions were driven by clinically meaningful factors including PLA2R-Ab titer, eGFR, age, and serum albumin, supporting model transparency and credibility. Subgroup analysis based on the 2.25 RU/mL cutoff showed distinct clinical implications. In the low-titer subgroup (≤ 2.25 RU/mL), the model yielded high specificity and negative predictive value to support risk stratification judgment. In the high-titer subgroup (> 2.25 RU/mL), the model achieved higher sensitivity and AUC, improving detection of potential false-negative PLA2R-associated MN. However, the low negative predictive value in this subgroup indicates that a negative model prediction cannot exclude MN, and biopsy remains warranted to avoid missed diagnoses.

Our study has notable strengths including a large sample size, strict validation framework, full model interpretability, and end-to-end clinical translation; all predictors are routinely available in clinical practice; the freely accessible web application enabled real-world implementation; and the clinical acceptance survey confirmed strong preference for this noninvasive tool among both physicians and patients. Several limitations should be acknowledged. First, this retrospective study requires prospective validation, and external validation was conducted in only a single geographic region, which may limit the generalizability of the findings. Second, novel biomarkers, including anti-THSD7A and anti-NELL-1 antibodies, were not incorporated into the model (34, 35). Additionally, although anti-PLA2R-IgG4 subtype had better predictive performance than total PLA2R antibody in previous reports, the corresponding detection reagents are unavailable in our center; thus, relevant serological data could not be obtained for analysis. Third, only diabetic nephropathy (DN), minimal change disease (MCD), and IgA nephropathy (IgAN) were included as non-MN controls because they represent the most common differential diagnoses of PLA2R-Ab-negative MN in routine clinical practice. Other proteinuric glomerular diseases were not included, and this limited disease spectrum may further restrict the model’s performance in broader clinical populations. Fourth, the 2.25 RU/mL inflection point identified by the restricted cubic spline analysis has not been externally validated in independent prospective cohorts, and its clinical applicability therefore remains to be established. In addition, the convenience sampling strategy used in the clinical acceptance survey may have reduced the representativeness of the respondents. Future studies should include prospective multicenter validation, incorporation of emerging autoantibody biomarkers to improve risk reclassification performance, evaluation of the model as an auxiliary tool for pre-biopsy clinical risk stratification, and independent validation of the 2.25 RU/mL inflection point in cohorts with a broader spectrum of control diseases.

## 5 Conclusion

We developed and validated an interpretable ML model for identifying PLA2R-Ab-negative membranous nephropathy. The model shows high risk stratification accuracy and specificity, with clear interpretability. It can act as a noninvasive auxiliary tool for risk stratification among PLA2R-Ab-negative proteinuric patients with suspected MN.

## Data Availability

The raw data supporting the conclusions of this article will be made available by the authors, without undue reservation.
