# Supplementary material for: Risk prediction of PLA2R-Ab-negative membranous nephropathy: an interpretable multicenter machine learning model
Source: Front Immunol. 2026 Sep 1;17:1906174. doi: 10.3389/fimmu.2026.1906174 (PMC13574958; doi:10.3389/fimmu.2026.1906174)
Supplement: Supplementary file 1 [file Table1.docx]

Supplementary Material

# 1 Supplementary Figures

**Supplementary Figure S1.** **Spearman correlation heat map of core clinical and laboratory features.** Pairwise Spearman correlation coefficients were calculated for continuous features, with features having a correlation >0.7 removed to reduce redundancy.

**Supplementary Figure S2. ROC curves of five-fold cross-validation for the final VotingSoft model.** Five cross-validation folds yielded AUC values of 0.879, 0.868, 0.900, 0.918, and 0.825, with a mean AUC of 0.878 ± 0.031 (95% CI: 0.850–0.903); all curves deviated significantly from the random classifier, confirming consistent model performance.

**Supplementary Figure S3. Comparison of diagnostic performance between the final model and standalone PLA2R-Ab.** Panel (a) shows ROC curves with the model achieving an AUC of 0.894, outperforming standalone PLA2R-Ab (AUC=0.738); Panel (b) presents DCA curves demonstrating superior net clinical benefit of the model across threshold probabilities (0.1–0.9); at the optimal threshold (0.2), the model achieved an NRI of −0.052 (95% CI: −0.122–0.022).

**Supplementary Figure S4. Web-based application interface for clinical translation of the PLA2R-Ab-negative MN diagnostic model.** Clinicians input 13 routinely available clinical/laboratory features to obtain instantaneous MN predicted probability and feature contribution breakdown consistent with SHAP analysis.
